# Assessment of Knowledge and Awareness Regarding Coronary Artery Disease Risk Factors Among the Saudi Arabian Population: A Cross-Sectional Study

**DOI:** 10.7759/cureus.52299

**Published:** 2024-01-15

**Authors:** Eman A Elsheikh, Osama H Alqahtani, Haneen M Aljedani, Saleh M AlKulayb, Omar M Bamousa, Rana M Althobaiti, Lama A Alharbi, Mohammed M Alqahtani, Osama F Al-Amri, Hadi S Alyami

**Affiliations:** 1 Internal Medicine, King Faisal University, Hofuf, SAU; 2 General Practice, Maternity and Children Hospital, Dammam, SAU; 3 General Practice, Ibn Sina National College for Medical Studies, Jeddah, SAU; 4 General Practice, Najran University, Najran, SAU; 5 General Practice, King Abdulaziz University, Jeddah, SAU; 6 General Practice, Taif University, Taif, SAU; 7 General Practice, Qassim University, Qassim, SAU; 8 General Practice, King Faisal University, Hofuf, SAU

**Keywords:** knowledge assessment, public awareness, obesity, cardiovascular disease, coronary artery disease

## Abstract

Background

Coronary artery disease constitutes a critical public health issue due to its widespread prevalence, placing a significant burden on healthcare systems and posing considerable challenges to the well-being of the population. Limited recent data on coronary artery disease awareness in Saudi Arabia underscores the need for updated information to inform preventive programs.

Methodology

A cross-sectional questionnaire survey was conducted from August 2021 to October 2022, targeting individuals aged 18 and above in Saudi Arabia. The online survey collected demographic data and assessed knowledge and awareness of coronary artery disease risk factors. A scoring system categorized participants as possessing high, medium, or low levels of awareness and knowledge. Data analysis involved a multivariable regression approach to explore associations.

Results

The study included a total of 1,409 participants, representing a diverse demographic distribution. Knowledge scores revealed 70.3% high, 27.4% medium, and 2.3% low levels, while awareness scores showed 59.9% high, 32.9% medium, and 7.2% low levels. The study identified significant associations between knowledge scores and gender (p = 0.003) and age (p = 0.001). Similarly, awareness scores demonstrated associations with gender (p = 0.001), age (p = 0.001), marital status (p = 0.003), educational qualification (p = 0.036), and occupation (p = 0.001). These findings underscore the multifaceted nature of factors influencing awareness and knowledge levels.

Conclusions

The study highlights a strong foundation of knowledge and awareness among the Saudi population regarding coronary artery disease risk factors, indicating a positive starting point for preventive initiatives. However, targeted programs addressing regional variations and enhancing knowledge are recommended to improve early detection and treatment of coronary artery disease risk factors.

## Introduction

Coronary artery disease poses a significant cardiovascular risk in Saudi Arabia, ranking among the leading causes of mortality for individuals aged 35 and older [1]. Globally, inadequate awareness of risk factors such as smoking, obesity, and physical inactivity contributes to a rise in premature coronary artery disease-related deaths, reaching 40% in East Asia and 27% in South Asia over the past two decades [2].

While coronary artery disease mortality rates have globally declined, it remains a noteworthy cause of death for adults aged above 35 in the Middle East, accounting for 13.4% [3]. Recent data on coronary artery disease mortality rates in Saudi Arabia are scarce, with the last update in 2004 indicating a 5.5% mortality rate between the ages of 30 and 70 [3].

Studies from Hail revealed varying awareness levels of coronary artery disease risk factors, with notable percentages for TV watching, smoking, lack of physical activity, and family history of coronary artery disease. However, awareness was lower for factors such as diabetes, family history of hypertension, and hyperlipidemia [4].

A European study reported stress as the most frequently cited coronary artery disease risk factor (63.3%), with gender differences less pronounced. Obesity was more commonly reported by females while smoking, alcohol consumption, and lack of exercise were more likely reported by males. Symptoms of heart attacks were well identified, but dyspnea, unusual fatigue, dizziness, and weakness showed lower recognition, with nausea and palpitations reported more frequently by females [5].

The study by Albadrani et al. from Qassim emphasized a lack of knowledge regarding coronary artery disease risk factors, despite variations in awareness levels [1]. Lower identification of warning symptoms increases the risk of delayed medical intervention, elevating morbidity and mortality rates. This study aims to assess the baseline knowledge in Saudi Arabia, informing the development of tailored prevention programs and reducing the burden of coronary artery disease.

## Materials and methods

Study design

This cross-sectional questionnaire survey, conducted in Saudi Arabia from August 2021 to October 2022, aimed to investigate the level of awareness regarding coronary artery disease risk factors among the Saudi Arabian population.

Study participants

An online survey questionnaire was distributed to participants selected through volunteer sampling. The survey, targeting individuals aged 18 years and above, had no sex restrictions. The questionnaire was disseminated through social media platforms and email. Participants meeting the inclusion criteria were aged 18 years and above and residing in Saudi Arabia. Exclusion criteria included individuals below 18 years old, those residing outside Saudi Arabia, healthcare-related professionals and students, individuals with a known history of coronary artery disease, and non-Arabic/non-English language speakers.

Study instrument

The questionnaire consisted of two sections. The first collected demographic data, while the second assessed knowledge and awareness of coronary artery disease risk factors. For this study, knowledge refers to the understanding and familiarity participants have about specific risk factors associated with coronary artery disease, such as smoking, obesity, and lack of physical activity. On the other hand, awareness in our study is defined as the recognition and consciousness participants have regarding the broader concepts of coronary artery disease, encompassing its symptoms and preventive measures.

The survey instrument was developed based on established measures, and its reliability and validity were ensured via a thorough review process. To assess questionnaire simplicity and study feasibility, a pilot test involving 20 individuals was conducted, with pilot study data excluded from the final analysis.

A scoring system was applied to the 35 statements used for assessment, with 15 statements measuring awareness and 20 statements measuring knowledge. Correct answers received one point, while incorrect answers or “I don't know” responses received zero points. Scoring categories were defined as follows: Awareness Assessment Scoring (11-15 for high awareness, 6-10 for medium awareness, and ≤5 for low awareness) and Knowledge Assessment Scoring (15-20 for high knowledge, 8-14 for medium knowledge, and ≤7 for low knowledge).

Data analysis

Data analysis for this study was meticulously executed using SPSS version 24 (IBM Corp., Armonk, NY, USA). To elucidate complex relationships between variables, a multivariable regression approach with a stepwise technique was employed. This methodology enabled the exploration of intricate associations, considering multiple independent variables and adjusting for potential confounding factors. The stepwise technique facilitated a systematic inclusion or exclusion of variables, enhancing the precision of the analysis.

Ethical considerations

The study obtained approval from King Faisal University (reference number: 2023-1300), ensuring adherence to ethical standards. Informed consent procedures were meticulously implemented, providing participants with comprehensive details about the study objectives, potential risks, and confidentiality measures. Participants were assured of their autonomy to withdraw at any stage without repercussion.

## Results

Sociodemographic characteristics

The study encompassed 1,409 participants, representing a diverse demographic distribution. Among them, 731 (51.9%) were females, and 678 (48.1%) were males. A majority of the participants (97.9%) were Saudi nationals, with only 30 (2.1%) participants being non-Saudi. Marital status indicated 719 (51.0%) single and 651 (49.0%) married participants. Age distribution revealed 617 (43.8%) individuals aged 20-30 years, 232 (16.5%) aged 31-40 years, and 226 (16.0%) aged 41-50 years. Occupationally, 577 (40.9%) identified as students, and 500 (35.5%) as employees. Regarding educational qualifications, 809 (57.4%) participants had a bachelor’s level as their highest qualification, followed by 323 (22.9%) at the secondary level (Table 1).

**Table 1:** Demographic profile and educational background of participants. Percentages are calculated based on the total number of participants (N = 1,409).

Variable		Frequency	Percentage
Age (years)	<20	175	12.4
	20–30	617	43.8
	31–40	232	16.5
	41–50	225	16.0
	51–60	119	8.4
	>60	41	2.9
Gender	Male	678	48.1
	Female	731	51.9
Nationality	Saudi	1,379	97.9
	Non-Saudi	30	2.1
Marital status	Single	719	51.0
	Married	651	46.2
	Divorced	25	1.8
	Widowed	14	1.0
Educational qualification	Elementary school	10	7.0
	Middle school	36	2.6
	High school	323	22.9
	Diploma	146	10.4
	Bachelor’s degree	807	57.3
	Master’s degree	66	4.7
	Doctorate	21	1.5
Working status	Employee	502	35.6
	Student	578	41.0
	Not currently employed	329	23.3

Knowledge and awareness scores

In the realm of knowledge and awareness scores, 990 (70.3%) participants exhibited a high level of knowledge, while 386 (27.4%) participants demonstrated a medium level, and 33 (2.3%) participants had a low level. Regarding awareness, 845 (59.9%) participants reported a high level, 464 (32.9%) participants had a medium level, and 101 (7.2%) participants reported a low level of awareness.

Relationship between knowledge and awareness scores and sociodemographic characteristics

The analysis unveiled significant associations between knowledge scores and gender (p = 0.003) and age (p = 0.001). Knowledge scores were found to be similar for males and females. However, no significant associations were observed with nationality (p = 0.690), marital status (p = 0.264), educational qualification (p = 0.229), and occupation (Table 2).

**Table 2:** Exploring the relationship between sociodemographic characteristics and knowledge scores. Percentages presented in the table are calculated based on the total number of participants (N = 1,409) included in the analysis for exploring the relationship between sociodemographic characteristics and knowledge scores. P-values were calculated using statistical tests to assess the significance of relationships. Significant associations are indicated by p-values less than 0.05. The chi-square test was employed for categorical variables such as age, gender, nationality, marital status, educational qualification, and working status.

Variable		Knowledge score			Total	P-value
		Low	Medium	High		
Age (years)	<20	7 (21.9%)	73 (18.9%)	95 (9.6%)	175 (12.4%)	0.001
	20–30	16 (50.0%)	143 (37.0%)	458 (46.2%)	617 (43.8%)	
	31–40	6 (18.8%)	74 (19.2%)	152 (15.3%)	232 (16.5%)	
	41–50	1 (3.1%)	60 (15.5%)	164 (16.5%)	225 (16.0%)	
	51–60	2 (6.3%)	24 (6.2%)	93 (9.4%)	119 (8.4%)	
	>60	0 (0.0%)	12 (3.1%)	29 (2.9%)	41 (2.9%)	
Gender	Male	19 (59.4%)	158 (40.9%)	501 (50.6%)	678 (48.1%)	0.003
	Female	13 (40.6%)	228 (59.1%)	490 (49.4%)	731 (51.9%)	
Nationality	Saudi	32 (100.0%)	378 (97.9%)	969 (97.8%)	1,379 (97.9%)	0.690
	Non-Saudi	0 (0.0%)	8 (2.1%)	22 (2.2%)	30 (2.1%)	
Marital status	Single	8 (25.0%)	181 (46.9%)	462 (46.6%)	651 (46.2%)	0.264
	Married	23 (71.9%)	195 (50.5%)	501 (50.6%)	719 (51.0%)	
	Divorced	1 (3.1%)	5 (1.3%)	19 (1.9%)	25 (1.8%)	
	Widowed	0 (0.0%)	5 (1.3%)	9 (0.9%)	14 (1.0%)	
Educational qualification	Elementary school	0 (0.0%)	3 (0.8%)	7 (0.7%)	10 (0.7%)	0.229
	Middle school	1 (3.1%)	10 (2.6%)	25 (2.5%)	36 (2.6%)	
	High school	10 (31.3%)	104 (26.9%)	209 (21.1%)	323 (22.9%)	
	Diploma	4 (12.5%)	47 (12.2%)	95 (9.6%)	146 (10.4%)	
	Bachelor’s degree	16 (50.0%)	206 (53.4%)	585 (59.0%)	807 (57.3%)	
	Master’s degree	0 (0.0%)	12 (3.1%)	54 (5.4%)	66 (4.7%)	
	Doctorate	1 (3.1%)	4 (1.0%)	16 (1.6%)	21 (1.5%)	
Working status	Employee	10 (31.3%)	132 (34.2%)	360 (36.3%)	502 (35.6%)	0.145
	Student	5 (15.6%)	106 (27.5%)	218 (22.0%)	329 (23.3%)	
	Not employed	17 (53.1%)	148 (38.3%)	413 (41.7%)	578 (41.0%)	

Awareness scores exhibited significant associations with gender (p = 0.001), age (p = 0.001), marital status (p = 0.003), educational qualification (p = 0.036), and occupation (p = 0.001). Nevertheless, no significant association was found with nationality (Table 3).

**Table 3:** Exploring the relationship between sociodemographic characteristics and awareness scores. Percentages presented in the table are calculated based on the total number of participants (N = 1,409) included in the analysis for exploring the relationship between sociodemographic characteristics and awareness scores. P-values were calculated using statistical tests to assess the significance of relationships. Significant associations are indicated by p-values less than 0.05. The chi-square test was employed for categorical variables such as age, gender, nationality, marital status, educational qualification, and working status.

Variable		Awareness score			Total	P-value
		Low	Medium	High		
Age (years)	<20	29 (28.7%)	79 (17.0%)	67 (7.9%)	175 (12.4%)	0.001
	20–30	43 (42.6%)	184 (39.7%)	390 (46.2%)	617 (43.8%)	
	31–40	13 (12.9%)	80 (17.2%)	139 (16.5%)	232 (16.5%)	
	41–50	10 (9.9%)	72 (15.5%)	143 (16.9%)	225 (16.0%)	
	51–60	3 (3.0%)	32 (6.9%)	84 (10.0%)	119 (8.4%)	
	>60	3 (3.0%)	17 (3.7%)	21 (2.50%)	41 (2.90%)	
Gender	Male	51 (50.5%)	184 (39.7%)	443 (52.5%)	678 (48.1%)	0.001
	Female	50 (49.5%)	280 (60.3%)	401 (47.5%)	731 (51.9%)	
Nationality	Saudi	98 (97.0%)	454 (97.8%)	827 (98.0%)	1,379 (97.9%)	0.820
	Non-Saudi	3 (3.0%)	10 (2.2%)	17 (2.0%)	30 (2.1%)	
Marital status	Single	29 (28.7%)	212 (45.7%)	410 (48.6%)	651 (46.2%)	0.003
	Married	70 (69.3%)	233 (50.2%)	416 (49.3%)	719 (51.0%)	
	Divorced	1 (1.0%)	13 (2.8%)	11 (1.3%)	25 (1.8%)	
	Widowed	1 (1.0%)	6 (1.3%)	7 (0.8%)	14 (1.0%)	
Educational qualification	Elementary school	1 (1.0%)	4 (0.9%)	5 (0.6%)	10 (0.7%)	0.036
	Middle school	4 (4.0%)	16 (3.4%)	16 (1.9%)	36 (2.6%)	
	High school	32 (31.7%)	121 (26.1%)	170 (20.1%)	323 (22.9%)	
	Diploma	11 (10.9%)	52 (11.2%)	83 (9.8%)	146 (10.4%)	
	Bachelor’s degree	50 (49.5%)	250 (53.9%)	507 (60.1%)	807 (57.3%)	
	Master’s degree	2 (2.0%)	16 (3.4%)	48 (5.7%)	66 (4.7%)	
	Doctorate	1 (1.0%)	5 (1.1%)	15 (1.80%)	21 (1.5%)	
Working status	Employee	30 (29.7%)	144 (31.0%)	328 (38.9%)	502 (35.6%)	0.001
	Student	52 (51.5%)	185 (39.9%)	341 (40.4%)	578 (41.0%)	
	Not employed	19 (18.8%)	135 (29.1%)	175 (20.7%)	329 (23.3%)	

## Discussion

Coronary artery disease remains a significant cardiovascular concern, affecting nearly half of middle-aged men and about one-third of middle-aged women in developed countries [6,7]. Despite a clear reduction in coronary heart disease-associated mortality, coronary artery disease remains a major cause of death in adults aged over 35 years [8]. This study aimed to assess the knowledge and awareness levels of coronary artery disease risk factors among the population in Saudi Arabia, recognizing the critical role of knowledge and awareness in disease prevention that extends beyond healthcare professionals to reach the general public [9].

Our findings revealed that 70.3% of participants reported a high level of knowledge regarding coronary artery disease risk factors, while 27.4% had a medium level, and only 2.3% exhibited a low level of knowledge. Similar results were reported in the southern region of Saudi Arabia, where 51.3% had good knowledge, 27.4% had moderate knowledge, and 21.3% had poor knowledge [10]. However, in Riyadh Province of Saudi Arabia, a study reported poor knowledge among respondents regarding both coronary artery disease risk factors and preventive measures [11]. Likewise, in Tabuk City, Saudi Arabia, there was a prevalence of poor knowledge regarding significant coronary artery disease risk factors [12]. In Bali City, Indonesia, 44.7% demonstrated good knowledge, 38.4% had average knowledge, and 16.9% had poor knowledge of coronary artery disease risk factors [13]. These variations underscore the need for targeted educational efforts across regions.

Regarding awareness scores, in our study, 59.9% of participants reported a high level of awareness, 32.9% a medium level, and only 7.2% a low level of awareness. In Bisha, 87.1% were aware of coronary artery disease and preventive measures [11]. However, in Tabuk City, only 8.16% were fully aware of all five key modifiable risk factors for heart disease [12]. In Taif City, 68.5% had good awareness, 29.4% had fair awareness, and only 2.1% had poor awareness [13]. These findings emphasize the need for targeted awareness campaigns tailored to specific regions.

Analyzing the association between knowledge scores and sociodemographic characteristics in our study, we found a significant association between knowledge scores and gender and age, in contrast to another study that reported significant associations with marital status, education level, and work [11]. Likewise, gender differences regarding knowledge about coronary artery disease risk factors were reported as insignificant in another study [13].

Concerning awareness scores, our study identified significant associations with gender, age, marital status, educational qualification, and occupation, aligning with another study where awareness was associated with occupation and educational level [12]. Results from Taif City indicated significant associations with age, educational level, and marital status, but not gender or employment status [13].

Despite the valuable insights gained from this study, certain limitations should be acknowledged. First, the data collection was reliant on self-reported information, introducing the possibility of recall bias and potential inaccuracies in participants’ responses. Additionally, the study’s cross-sectional design limits our ability to establish causation and temporal changes in knowledge and awareness levels cannot be fully captured. The sample primarily represents specific regions in Saudi Arabia, potentially limiting the generalizability of findings to the entire population [14,15].

## Conclusions

Our study unveils a predominantly high level of knowledge and awareness among participants regarding coronary artery disease risk factors, surpassing findings from comparable studies. This positive outcome suggests a foundational understanding among the Saudi population, offering an optimistic starting point for further initiatives. Nevertheless, recognizing areas for improvement remains crucial. We advocate for the development of targeted programs aimed at enhancing knowledge, with the overarching goal of bolstering early detection and treatment of risk factors. By continually refining public awareness, we anticipate a tangible reduction in the overall risk of coronary artery disease, contributing to a healthier and more informed population.
